# The effects of serum lipids on the *in vitro* activity of lumefantrine and atovaquone against *Plasmodium falciparum*

**DOI:** 10.1186/1475-2875-11-177

**Published:** 2012-05-28

**Authors:** Kesinee Chotivanich, Mathirut Mungthin, Ronnatrai Ruengweerayuth, Rachanee Udomsangpetch, Arjen M Dondorp, Pratap Singhasivanon, Sasithon Pukrittayakamee, Nicholas J White

**Affiliations:** 1 MORU, Faculty of Tropical Medicine, Mahidol University, 420/6 Rajvithi Rd, Bangkok, 10400, Thailand; 2 Department of Parasitology, Phramongkutklao College of Medicine, 315 Rajvithi Rd, Bangkok, 10400, Thailand; 3 Mae Sot Hospital, Tak, Thailand; 4 Department of Pathobiology, Faculty of Science, Mahidol University, Bangkok, Thailand; 5 Centre for Tropical Medicine, Churchill Hospital. University of Oxford, Oxford, UK

**Keywords:** Malaria, Anti-malarial drugs, *In vitro*-susceptibility

## Abstract

**Background:**

Lumefantrine and atovaquone are highly lipophilic anti-malarial drugs. As a consequence absorption is increased when the drugs are taken together with a fatty meal, but the free fraction of active drug decreases in the presence of triglyceride-rich plasma lipoproteins. In this study, the consequences of lipidaemia on anti-malarial drug efficacy were assessed *in vitro*.

**Methods:**

Serum was obtained from non-immune volunteers under fasting conditions and after ingestion of a high fat meal and used in standard *Plasmodium falciparum* in-vitro susceptibility assays. Anti-malarial drugs, including lumefantrine, atovaquone and chloroquine in five-fold dilutions (range 0.05 ng/ml – 1 ug/mL) were diluted in culture medium supplemented with fasting or post-prandial 10% donor serum. The in-vitro drug susceptibility of parasite isolates was determined using the ^3^H-hypoxanthine uptake inhibition method and expressed as the concentration which gave 50% inhibition of hypoxanthine uptake (IC_50_).

**Results:**

Doubling plasma triglyceride concentrations (from 160 mg/dL to 320 mg/dL), resulted in an approximate doubling of the IC_50_ for lumefantrine (191 ng/mL to 465 ng/mL, P < 0.01) and a 20-fold increase in the IC_50_ for atovaquone (0.5 ng/mL to 12 ng/ml; P < 0.01). In contrast, susceptibility to the hydrophilic anti-malarial chloroquine did not change in relation to triglyceride content of the medium.

**Conclusions:**

Lipidaemia reduces the anti-malarial activity of lipophilic anti-malarial drugs. This is an important confounder in laboratory *in vitro* testing and it could have therapeutic relevance.

## Background

Malaria remains one of the most important diseases in the tropical world. The main threat for malaria control is the emergence and spread of insecticide resistance in vector mosquitoes and anti-malarial drug resistance in malaria parasites. Resistance of *Plasmodium falciparum* to chloroquine and sulphadoxine-pyrimethamine arose in SE Asia and has spread subsequently to Africa resulting in the deaths of millions of children [1,2]. Resistance of *Plasmodium vivax* to chloroquine and pyrimethamine has also been reported in many parts of the world [3-7]. An important driver for both the emergence and spread of anti-malarial drug resistance is underdosing of anti-malarials [8]. Underdosing in young children and pregnant women is common, because blood concentrations of several anti-malarial drugs are significantly lower compared to those in non-pregnant adults [9]. Deployment of substandard quality or counterfeit drugs is another important contributor, although difficult to quantify [10]. Pharmacokinetic variability is a major confounder resulting in low plasma concentrations despite taking the correct dose of a quality assured drug.

 Anti-malarial drugs bind variably to plasma constituents and blood cells. Partioning of drugs in the blood may affect their access to malaria parasites. Atovaquone and lumefantrine are both highly effective lipophilic anti-malarial drugs. They are used as fixed dose combinations as atovaquone-proguanil and artemether-lumefantrine, respectively. Atovaquone-proguanil is used mainly as a chemoprophylactic [11] and artemether-lumefantrine is the most widely used artemisinin combination treatment of uncomplicated falciparum malaria.

Atovaquone is a hydroxynaphthoquinone, with a partition coefficient (log P) of 5.1. When given alone it rapidly leads to drug resistance, since single point mutations in the cytochrome B gene of *P. falciparum* can confer up to a 10,000 fold reduction in sensitivity to the drug [12]. Atovaquone is, therefore, only deployed in combination with proguanil (Malarone ^R^), which has a synergistic effect with atovaquone and also provides some protection against the emergence of drug resistance.

Lumefantrine is an aryl-aminoalcohol, with a similar mechanism of action to that of quinine, mefloquine, and halofantrine. It is highly lipophilic with very high (98%) binding to lipoproteins and fat in plasma. Absorption of atovaquone and lumefantrine are both increased when the drugs are taken with fat, which is the current recommendation [13-16].

However, the increase in drug absorption with a fatty meal might be offset, at least temporarily, by a reduction in the free fraction of the plasma concentration of the drug since the fatty meal will increase the concentration of plasma lipids leading to increased drug partitioning to the plasma fraction. It is not known whether this has pharmacodynamic consequences. Whether the parasiticidal activity of these lipophilic anti-malarial drugs might be affected by the concentration of lipids present in the culture medium was therefore investigated.

## Methods

This study was approved by the Ethics committee of the Faculty of Tropical Medicine, Mahidol University (MUTU2006-032). Written informed consent forms were signed by the volunteers as well as patients before recruitment into this study.

### Serum preparation

Blood (20 mL) was taken from three healthy volunteer subjects (N = 3) after an overnight fast and also at 2 and 4 hours after consuming a meal containing more than 50g fat (a Mac Donald’s BigMac and a tall portion of French fries). Blood samples were centrifuged at 800 g for 5 minutes. Serum was collected, inactivated at 56°C in a water bath and kept at 30°C until use. Triglyceride levels in serum and in the culture medium were determined by an automated biochemical analyser (Cobas 501, Rocse. Switzerland).

### Preparation of drugs

Triplicate 96 well plates, pre-coated with anti-malarial drugs were prepared as follows: lumefantrine (1 mg/mL in linoleic acid: 100% ethanol, 1:1) and atovaquone (1 mg/mL in 100% ethanol) in five-fold dilutions (range 0.05 ng/ml – 1 μg/mL) as described previously [17,18]. Drugs were diluted in culture medium supplemented with fasting or post-prandial 10% donor serum. The hydrophilic anti-malarial chloroquine (range 0.05 ng/ml – 1 μg/mL) was used as a negative control.

### *In vitro* efficacy assessment

*Plasmodium falciparum* laboratory strain (TM267 from Thailand) and ten *P. falciparum* parasites isolated from patients who visited Mae Sot Hospital, Tak, Thailand, were cultured using standard procedures [19]. *In vitro* drug sensitivity testing also followed standard methods. Briefly, each well contained an aliquot (100 μL) of culture, and 100 μL of red cell suspension (final haematocrit 5%) with a 1% ring stage parasitaemia. Parasites were incubated at 37°C and 5% CO_2_ for 24 hours, after which 85 μL of medium was removed and refreshed by replacement of an equal volume of anti-malarial drug supplemented medium. ^3^H-hypoxanthine (stock = 1 millicurie/mL) was added to obtain a final dilution at 100 microcurie/mL. After 48 hours of further incubation, cells were harvested and incorporation of hypoxanthine was assessed in a beta-counter. The level of hypoxanthine uptake was determined as a measure of schizont development. The 50% inhibitory concentration (IC_50_), (the concentration which gave 50% inhibition of hypoxanthine uptake) was calculated from the sigmoid fitted curve, using Winnonlin computer software version 5.3 (Pharsight, USA). The IC_50_ values between experimental conditions were compared using the Mann- Whitney *U* Test. Correlations between the IC_50_ and triglyceride concentrations in the culture medium were calculated according to the methods of Pearson and Spearman.

## Results

**Figure 1:**
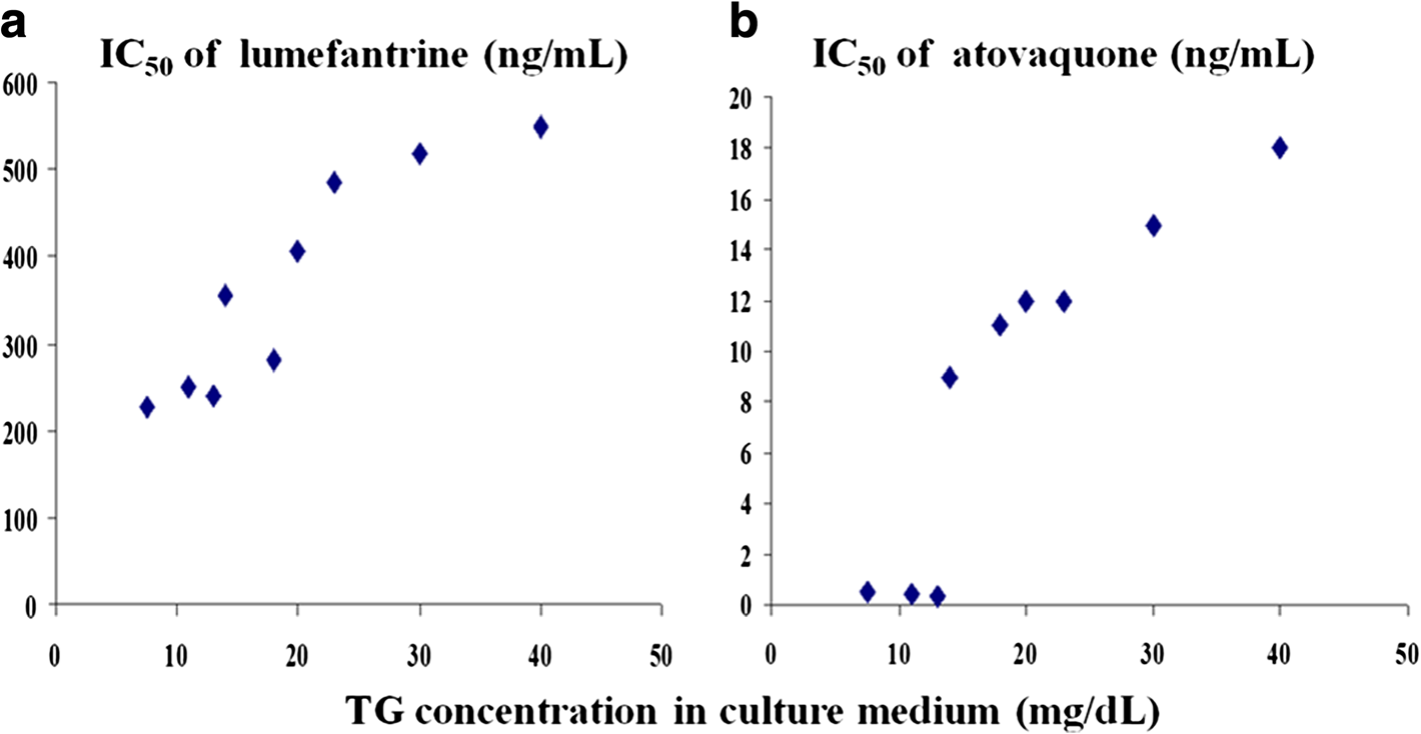
**In-vitro drug susceptibility of Thai laboratory strain (TM267) to lumefantrine and atovaquone under culture conditions with varying concentrations of triglycerides (TG) in the supplemented serum (10% v/v).** Figure **a**: IC_50_ for lumefantrine (ng/mL); figure **b**: IC_50_ for atovaquone (ng/mL).

The mean (SE) serum triglyceride levels (N = 3) were 106 (29) mg/dL when fasting, and 200 (15) and 377 (25) mg/dL at 2 and 4 hours after the high fat meal, respectively. Addition of post-prandial serum markedly reduced the *in vitro* activity of both drugs. There was an approximately linear correlation between the triglyceride concentrations of the added serum and the IC_50_ for both lumefantrine (R = 0.95, P < 0.01) and for atovaquone (R = 0.96, P < 0.01) (Figure 1). Ten parasite isolates were obtained for in-vitro drug susceptibility assessment. With a two fold increase in serum triglyceride concentrations (from 106 mg/dL to 200 mg/dL), there was an approximate two-fold increase in the IC_50_ for lumefantrine (191 ng/mL to 465 ng/mL, P < 0.01) and a 20-fold increase in the IC_50_ for atovaquone (0.5 ng/mL to 12 ng/ml; P < 0.01). In contrast, the parasiticidal efficacy of the hydrophilic drug chloroquine was unaffected by the triglyceride concentration of the serum added to the culture medium (Table 1).

**Table 1:** **50% Inhibition concentrations (IC**_**50)**_**(ng/mL) of lumefantrine, atovaquone and chloroquine under different culture conditions: using medium supplemented with 10% serum obtained from a fasting donor (‘fasting’) or obtained 2 hours after ingestion of a fatty meal (‘2** **hours’)**

**Drug**	**Serum used in culture medium**	**IC 50 (ng/mL)(Mean ± SD)**
Lumefantrine	fasting	191 ± 53
	2 hours	465 ± 34^*^
Atovaquone	fasting	0.5 ± 0.3
	2 hours	12 ± 6*
Chloroquine	fasting	94 ± 39
	2 hours	94 ± 14

Data of three replicate measurements of the same parasites (N = 10)

* _P<0.005_ when compared to fasting.

## Discussion

The *in vitro* anti-malarial drug activitiesof lumefantrine and atovaquone depend on the concentrations of lipid in the culture medium. The lack of any effect of post-prandial serum on the *in vitro* activity of chloroquine argues against any non-specific anti-malarial effect related to the high protein high fat meal. The decrease in anti-malarial drug activity of atovaquone and lumefantrine in the presence of triglyceride rich lipoproteins presumably reflects increased partitioning to this fraction and a reduced unbound fraction of the lipophilic drugs available to access the parasitized erythrocyte. Increased partitioning to the lipid fraction therefore reduced biological activity. Similar results have been reported for halofantrine, which is also highly lipophilic [20]. The administration of halofantrine with food increases its absorption, and may lead to excessively high plasma halofantrine concentration and cardiac toxicity [21]. Acute malaria also induces changes in plasma lipoprotein profiles [22]. These factors may affect the pharmacokinetic and the pharmacodynamic profiles of these lipophilic anti-malarial drugs.

Artemether-lumefantrine is highly effective treatment of multi-drug resistant falciparum malaria [23,24] and has become the most widely used ACT in the world. Both artemether and lumefantrine absorbtion are increased by fats although the effect on lumefantrine is greater. As a result it is recommended that lumefantrine should be taken together with fat, although a relatively small quantity (1.2 g) is necessary to achieve 90% of the maximum effect [16]. As the initial anti-parasitic effects are mediated mainly by the artemether component, these findings of reduced lumefantrine activity in lipaemic blood are unlikely to be of therapeutic relevance to the initial response. However anti-malarial efficacy during the terminal elimination phase, when plasma concentrations of the lumefantrine are low and patients resume their normal diet, may be affected by dietary fat intake. For atovaquone-proguanil, which is mainly used as a prophylactic drug, efficacy in hyperlipidaemic travellers might be compromised. The transient elevations in triglycerides that follow meals are unlikely to affect anti-malarial activity greatly. The reduction in anti-malarial activity would be greatest in patients with persistently elevated plasma triglycerides from conditions which are common in travelers such as hyperlipoproteinaemia, diabetes, and the metabolic syndrome. Patients receiving anti-retroviral drugs may also have sustained hypertriglyceridaemia and they too might have anti-malarial efficacy compromised. Conversely atorvastatin, which is widely used in the treatment of hypercholesterolaemia, has significant anti-malarial properties [25]. Further studies to determine the therapeutic relevance of these observations are warranted.

## Conclusions

*In vitro* tests are used to monitor anti-malarial drug susceptibility. These data suggest that post-prandial or hyperlipidaemic blood or serum samples may not be appropriate for *in vitro* testing of lipophilic anti-malarials. Lipidaemia may contribute to variance in susceptibility testing and confound assessments of resistance.

## Competing interests

The authors declare that they have no competing interests.

## Authors’ contributions

KC: designed and conducted the experiments, performed the data analysis and wrote manuscript. MM: technical support and data collection. RR and SP: patient recruitment and patient care, blood collection. RU: laboratory support and revised manuscript. AMD: data analysis and revised manuscript. PS: volunteer recruitment and blood collection. NJW: study design, data analysis and contributed to writing the manuscript. All authors have seen and approved this manuscript.
